# Quantitative investigation of diatom dispersion in lung tissue of confirmed drowning incidents

**DOI:** 10.1007/s00414-025-03441-1

**Published:** 2025-02-08

**Authors:** Dominik Hagen, Astrid Obermayer, Walter Stoiber, Peter Steinbacher, Jian Zhao, Fabio C. Monticelli, Stefan Pittner

**Affiliations:** 1 Dept. of Environment and Biodiversity, Paris-Lodron University of Salzburg, Salzburg, Austria; 2 Dept. of Forensic Medicine and Forensic Psychiatry, Salzburg, Austria; 3 Guangzhou Forensic Science Institute & Key Laboratory of Forensic Pathology, Ministry of Public Security, Guangzhou, China

**Keywords:** Diatoms, Drowning, L/D-ratio, SEM, Lung positions

## Abstract

The diagnosis of drowning represents a considerable challenge in forensic practice. Typically, it is made by excluding other potential causes of death while concurrently identifying key signs of drowning. However, in cases with a prolonged postmortem interval, these characteristic signs are often absent. To support drowning diagnoses, various supplementary methods have been introduced over time, prominently including qualitative and quantitative diatom tests. One such approach involves comparing diatom concentrations in the drowning medium (immersion water) and in the lung tissue to determine lung-to-drowning medium (L/D) ratios. L/D-ratios above 2 are thereby seen as safe indicators of drowning, as higher diatom concentrations in the lungs than in the drowning medium can only be explained by active water aspiration. Previous studies typically investigated diatom concentrations in the tip of the lung’s left superior lobe, as this region is thought to generally experience the lowest physiological pressure. However, no experimental data have yet confirmed the relevance of this in drowning incidents. It is also still unclear how diatoms are distributed within the lung during aspiration and how L/D-ratios could vary if samples from other lung regions are used for L/D-ratio calculations. To address these uncertainties, we conducted a study assessing diatom concentrations across seven positions from all pulmonary lobes in 25 drowning cases. Our aim was to identify the lung regions with the lowest probability of diatom uptake, thereby maximizing the reliability of L/D-ratios as a forensic tool. Our findings show that the left superior lung lobe is indeed the site of the lowest diatom accumulation and thus optimal for L/D-ratio related drowning diagnosis. Nonetheless, due to the higher sensitivity for diatom uptake, inclusion of other lung positions may also be recommended to strengthen diagnostic accuracy, especially in cases with generally low L/D-ratios.

**Supplementary Information:**

The online version contains supplementary material available at 10.1007/s00414-025-03441-1.

## Introduction

Since its first application by Revenstorf in 1904, the diatom test has evolved into a widely used standard technique in forensic expertise to find and/or support diagnosis in presumed drowning incidents [1]. The test is related to the ubiquitous presence of diatoms in almost every natural water body, and the unavoidable incorporation of diatoms through the aspiration of water during the drowning process. As long as circulation is active, diatoms are able to pass the alveolar-capillary membrane and reach peripheral organs via the bloodstream, suggesting that their presence in the liver and kidney can serve as an evidence for drowning [2, 3, 4]. Apart from that, their presence in lung tissue remains controversial due to the possibility of postmortal water infiltration into the lung via the respiratory tract [5–7]. The original diatom test was conducted by digesting the biological tissues with strong acids, followed by purification and deacidification of the produced pellet and light microscopical assessment of the acid stable diatom frustules. Since decades, the test has been criticized for its risk of false positivity due to contamination effects [8, 9]. More reliable results were recently achieved by quantitative analysis using the microwave digestion–vacuum filtration–automated scanning electron microscopy technique (MD-VF-Auto SEM) [10, 11] and a less elaborate modified version of this method from our lab [12]. Both methods combine microwave digestion followed by membrane filtration to enable maximal recovery of diatoms. Moreover, recent analysis focuses on comparing the diatom concentrations of lung tissue and the drowning medium to calculate L/D-ratios [13]. L/D-ratios above 1 already indicate for drowning with high probability, but if concentrations in the lung are found more than twice as high as those in the drowning medium (i.e. L/D-ratios above 2), this has been accepted as reliable proof of active water aspiration and consequently of true drowning. On the contrary, lower values may rather imply post mortal immersion, as passive water infiltration can at most result in equal diatom concentrations (L/D-ratio ≤ 1) [13, 14].

Tissue sampling has been almost exclusively confined to the tip of the left superior lung lobe, the presumed point of minimal pressure during a drowning process [15]. However, to date no experimental data exist to confirm this area as the optimal sampling site/position for diatom analyses and it remains unclear how diatoms are distributed in the lung in course of water aspiration, or whether other lung positions are potentially better suited for L/D-ratio calculations. To further clarify the distribution behavior of aspirated water and diatoms within the lung during the drowning process, we used our modified method of SEM-based diatom testing [12] to investigate diatom concentrations in tissue samples from seven different lung positions across all pulmonary lobes of 25 confirmed drowning cases. Results are discussed in terms of the deposition dynamics of diatoms in relation to anatomical conditions and their implications for the use of distinct lung positions for LD-ratios calculations in drowning diagnostics.

## Materials and methods

### Study cases and tissue sampling

A total of 25 cases (16 male, 9 female) with an average age of 56 years were included into the study. Drowning incidents occurred in local natural water bodies (rivers, lakes and ponds) in all seasons. Prior to the present examination, all cases were confirmed as drowning incidents by case investigation and/or autopsy, most of them presenting several of the classical distinct drowning signs such as emphysema aquosum, foam cone, foamy liquid in the airpassages, Paultauf’s spots, splenic anemia and liquid in the sphenoid sinuses (Svechnikov`s sign) (Table 1).

**Table 1:** Demographic data and autopsy findings of 25 confirmed drowning cases in chronological order; EA emphysema aquosum, FC foam cone, FL foamy liquid in air passages, PS Paltauf’s spots, SA splenic anemia, SS Svechnikov’s sign

case no.	gender	age	season	water body	drowning signs
**1**	female	64	summer	river	EA, FL, PS, SS
**2**	male	78	summer	pond	EA, FL, SA, SS
**3**	male	43	autumn	lake	EA, FC, SS
**4**	female	60	winter	lake	EA, FL, SA, SS
**5**	female	51	winter	river	EA, FC, PS
**6**	male	46	spring	river	EA, SS
**7**	male	63	autumn	lake	EA, FC, PS, SS,
**8**	male	18	autumn	river	EA, FC, PS
**9**	female	70	autumn	river	EA, FL, SS
**10**	female	17	summer	river	EA, FL, SS
**11**	male	62	summer	river	EA, FL, SS
**12**	male	60	autumn	stream	FL, SS
**13**	male	16	autumn	stream	EA, FL
**14**	male	81	autumn	pond	EA, FC, SS
**15**	female	61	summer	river	EA, LSC, SA, SS
**16**	male	74	summer	stream	EA, LSC, SA, SS
**17**	male	55	autumn	lake	EA
**18**	female	70	winter	lake	EA, LSC, SA
**19**	female	69	winter	river	EA, LSC, FL, SA
**20**	female	78	spring	stream	EA, LSC, SA, SS
**21**	male	62	spring	river	EA, FL, LSC, SS
**22**	male	47	spring	river	EA, FL, SA
**23**	male	20	autumn	river	SS
**24**	male	66	winter	n.a.	EA
**25**	male	66	winter	river	EA, FL, SA, SS

**Fig. 1:**
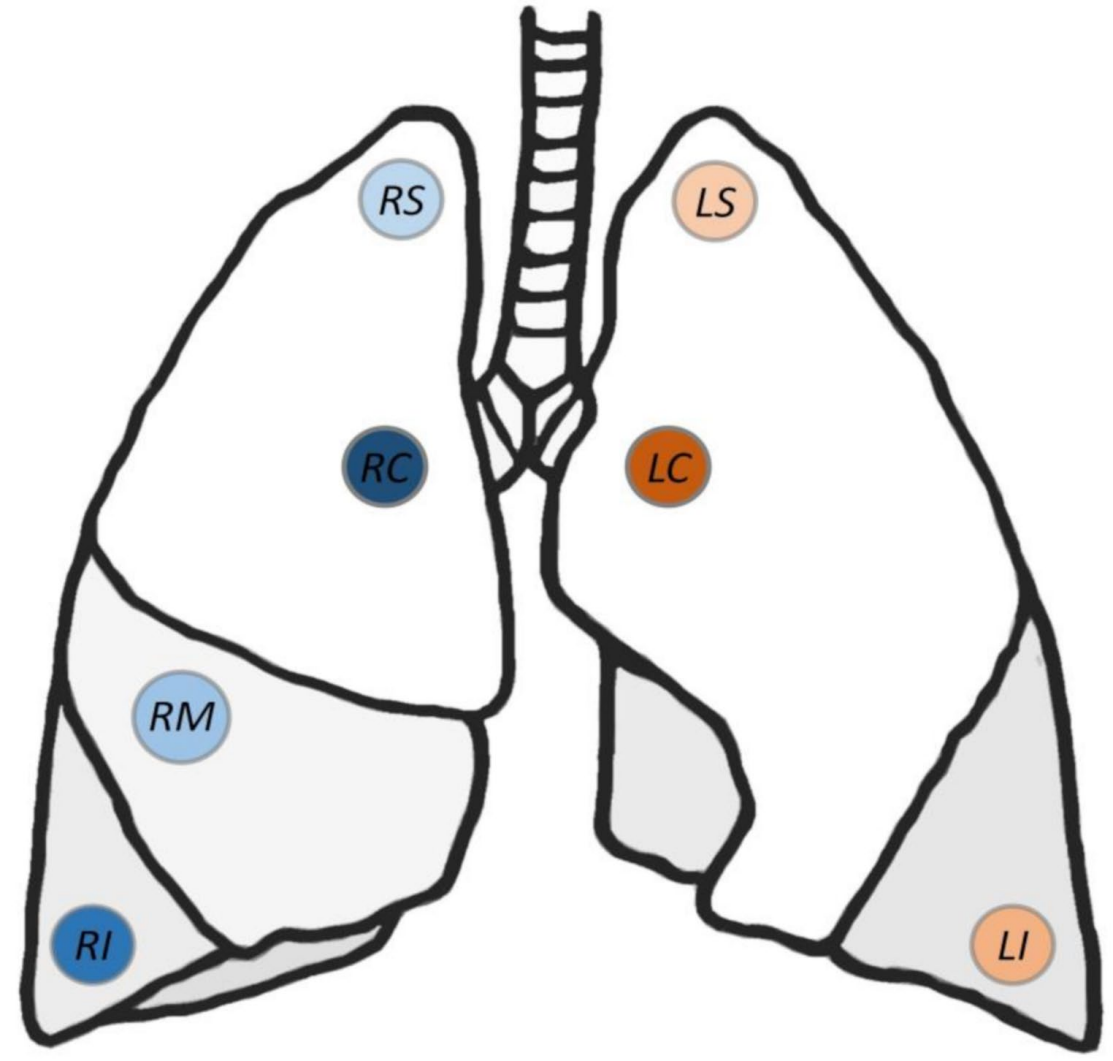
Sampling positions of the left and right lung. LS = left superior, LI = left inferior, LC = left central, RS = right superior, RM = right medial, RI = right inferior, RC = right central

Lung tissue samples of 25 drowning cases were collected according to respective standards during routine autopsies at the Department of Legal Medicine of the University of Salzburg [16] and were taken at the following seven positions: apical and central/bronchial regions of the right (RS, RC) and left superior lobe (LS, LC), right and left inferior lobe (RI, LI), and medial lobe of right lung (RM) (Fig. 1). At each position, at least 10 g of lung tissue was collected with thoroughly cleaned or disposable instruments to avoid any kind of cross-contamination and was stored in plastic cups at -20 °C until further investigation.

In 10 cases, reference samples of drowning water were secured by police upon body discovery and stored in light-tight plastic cups at 4 °C until further use. For these cases, L/D-ratios were calculated with each lung position’s respective diatom concentration to compare associated differences. On the same purpose, L/D-ratios of the 15 remaining cases without reference water samples were simulated in the following way: Given the thresholds specified above (i.e. ratios at or below 1 insufficient, ratios higher than 1 most likely drowning and higher than 2 reliable to confirm drowning), drowning water diatom concentrations were assumed such that the resulting L/D-ratios for each lung position ascended in steps of 0.15 in the range between 0.4 and 2.5 (Fig. 5).

### Sample processing and SEM analysis

All tissue samples were processed under sterile conditions. For each case 0.5–1.0 g tissue per lung position *(each time exactly weighed)* and depending on availability, 10 ml drowning medium were transferred into separate teflon-vessels of the Multiwave GO Plus microwave digestion system (Anton Paar). After adding 10 ml nitric acid (65% Carl Roth), the vessel-caps were screwed hand-tight and the digestion program was started. An initial 10-minute heating phase to 180 °C was followed by a 10 min stationary phase at this temperature and a 10-minute cooling phase to 70 °C. Subsequently, the caps were carefully unscrewed to enable gas outflow and the clear digestion solution was completely filtered through acid-stable polyvinylidenfluoride (PVDF) membranes (Ø = 1.0 cm, pore size 0.45 μm) using a custom-built automated syringe pump system [12]. Membranes were then deacidified with ultrapure water, decalcified with pure ethanol and dried in an incubator at 40 °C.

Dry membranes were sputter-coated with gold and analysed in a Philips/FEI XL30 ESEM scanning electron microscope refitted with Point Electronic software. To represent the whole membrane, 69 images per sample were manually scanned at 15 kV and a magnification of 500x following a distinct scatter-pattern with uniformly distributed coordinates [12].

### Data processing and statistical analysis

Diatoms of each image-set were counted using ImageJ software (1.54f). Following the adapted procedure of total population estimation [12], diatom counts were subsequently projected to total numbers of diatoms per filter and further converted into values of diatoms-per-g of tissue and diatoms-per-ml drowning water, in order to enable comparison between the individual lung position’s diatom concentrations and L/D-ratio calculations.

Nonparametric Friedman’s-ANOVA and corresponding post hoc tests (pairwise comparison and effect size calculation) were performed for statistical analyses using Microsoft Excel and SPSS 29 software, regarding *p* < 0.05 as statistically significant.

## Results

### General findings and statistical evaluation

**Fig. 2:**
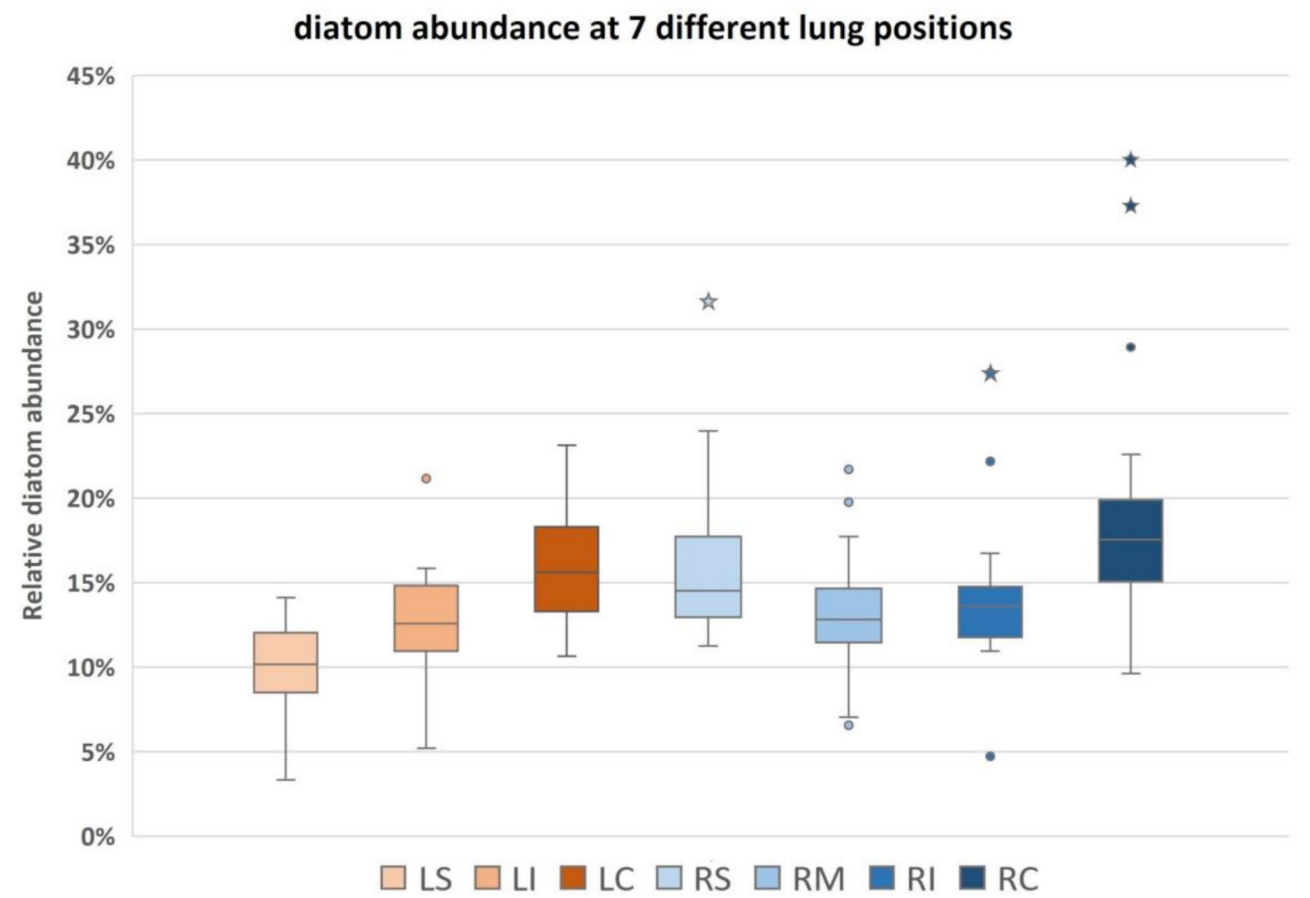
Relative diatom distribution illustrating minima, maxima, upper and lower quartiles and median values in seven different lung positions of 25 drowning cases, spots and asterisks indicate normal and extreme outliers. LS left superior, LI left inferior, LC left central, RS right superior, RM right medial, RI right inferior, RC right central

Owing to seasonal change of diatom abundance and different rheological conditions at the drowning sites (standing or running waters), diatom concentrations varied largely between the individual cases. As expected, cases that occurred during summer and autumn seasons mostly showed higher concentrations compared to winter and spring cases. To enable relative comparison between all cases, diatoms per-g-values of each case were recalculated as percentages of the total diatom count of all 7 lung positions, in the following abbreviatedly referred to as “%-of-total-count” (Fig. 2). (Overview of related data shown in Supplementary table S1 and Figure S1).

Measured in %-of-total-count, comparison of all tested lung positions over all cases revealed that in half of the cases, position LS displays values between 8.5 and 12%, with a minimum of 3.3%, a maximum of 14%, and a median of 10.1%, thus being the site of lowest diatom concentration among all tested lung positions. Second lowest concentrations were displayed at the positions LI, RM and RI, with values varying between 10.9 and 14.8% in 50% of the cases. Although these three positions presented similar median values (12.6%, 12.8% and 13.6%), they showed more marked differences in their minima and maxima. Respective values were at 5.2% and 15.8% in LI, whereas RM exhibited a slightly broader range of 7% and 17.7% and in RI ranging between 10.9% and 16.7%, although with an extreme outlier. Samples of position RS presented a clearly higher diatom content than those of the contralateral LS position, with 50% of the cases displaying values between 12.9% and 17.7%, at a median value of 14.5% and a minimum and maximum of 11.3% and 24%. Highest concentrations were displayed by the central positions LC (median at 15.6%, 50% of data between 13.3 and 18.3%) and RC (median at 17.5%, 50% of data between 15.1 and 19.9%) at minima and maxima of 11.3% and 24% in LC, and 9.6% and 22.6% in RC, the latter presenting two extreme outliers above the maximum.

Comparison of the investigated lung position’s diatom concentrations with Friedman’s ANOVA confirmed significant differences between the positions at a *p* < 0.001 (Table 2).

**Table 2:** Friedman’s two-way analysis of variance by ranks

total *N*	chi-square	degree of freedom	asymptotic sig.
25	66.549	6	*p* < 0.001

**Fig. 3:**
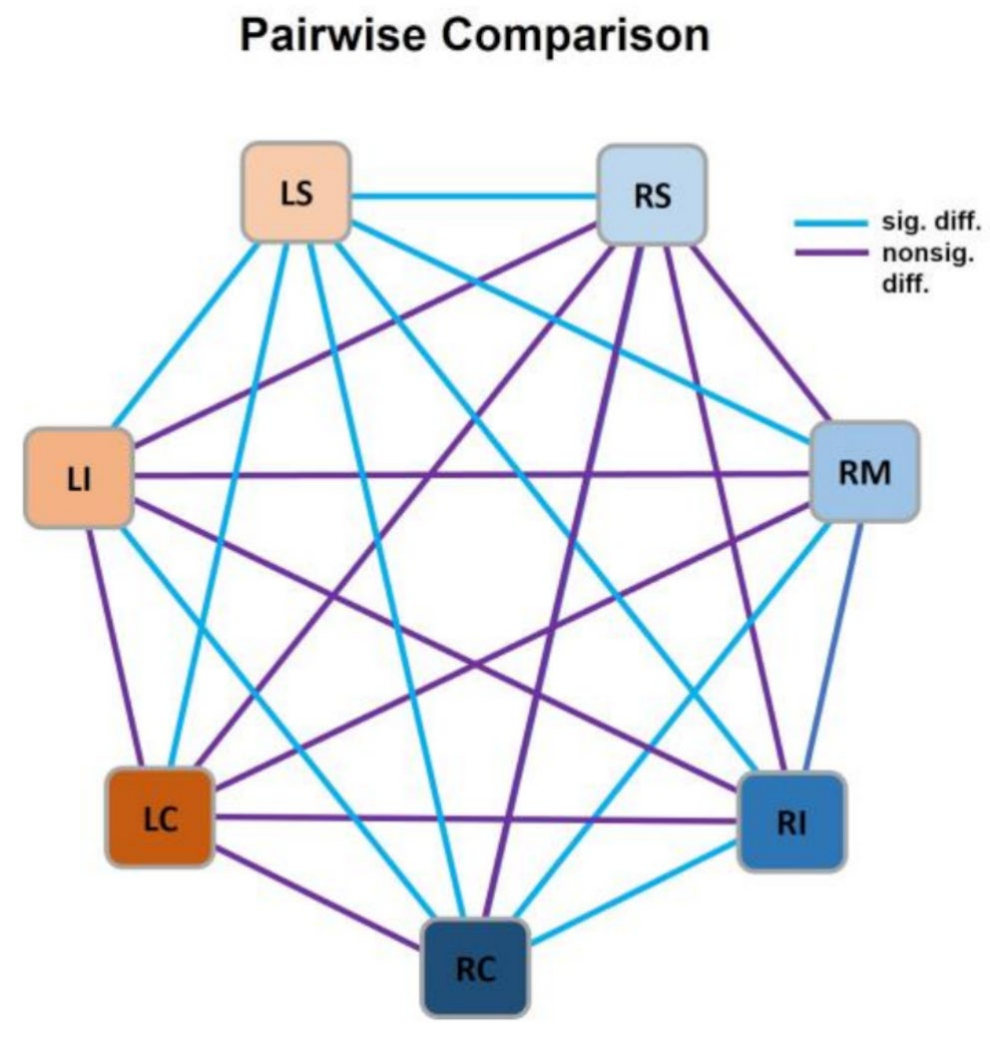
Pairwise comparison of median rank values; blue lines indicate significant differences between the connected positions, purple lines indicate no significant differences between the connected positions

Additional post-hoc analyses by pairwise comparison of individual positions were performed to investigate these differences in diatom accumulation in more detail (results summarized in Table 3; Fig. 3). Position LS showed significant differences to all other positions, adjusted significance-values being at *p* = 0.014 for comparison with LI, at *p* = 0.007 for comparison with RM, at *p* = 0.001 for comparison with RI, and at *p* < 0.001 for all other comparisons. Cohen’s effect size classification resulted in medium intensity when LS is compared to LI, RM and RI, and high intensity when compared to RS, LC and RC. Significant differences of medium effect size intensity were also found for the comparisons of position RC to positions LI, RM and RI, and for the comparison of LI to LC. All other tested comparisons showed no significant difference.

**Table 3:** Pairwise comparison of median rank values of all examined lung positions with adjusted significance levels (Bonferroni correction) and effect size classification (Cohen 1992)

position 1- position 2	adj. sig.	*r*	effect size
LS-LI	= 0.014	0.416	medium
LS-RM	= 0.007	0.440	medium
LS-RI	= 0.001	0.496	medium
LS-RS	< 0.001	0.624	high
LS-LC	< 0.001	0.792	high
LS-RC	< 0.001	0.872	high
LI-RM	= 1.000	0.024	
LI-RI	= 1.000	0.080	
LI-RS	= 1.000	0.208	
LI-LC	= 0.044	0.376	medium
LI-RC	= 0.004	0.456	medium
RM-RI	= 1.000	0.056	
RM-RS	= 1.000	0.184	
RM-LC	= 0.083	0.352	
RM-RC	= 0.009	0.432	medium
RI-RS	= 1.000	0.128	
RI-LC	= 0.324	0.296	
RI-RC	= 0.044	0.376	medium
RS-LC	= 1.000	0.168	
RS-RC	= 0.891	0.248	
LC-RC	= 1.000	0.080	

### Deviations of each lung position’s diatom concentration to the minimal measured concentrations

**Fig. 4:**
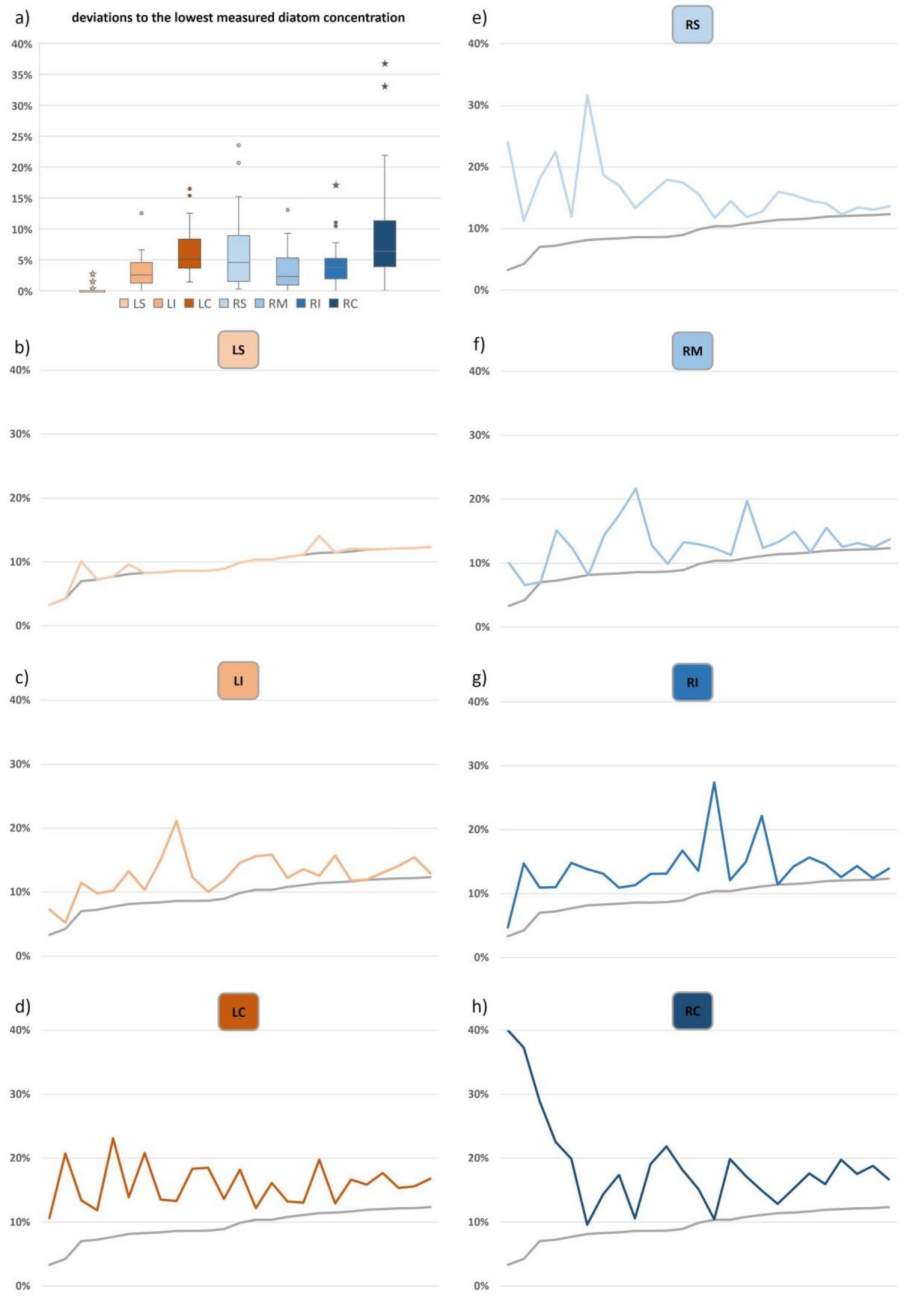
Deviations of positional diatom concentrations from the minimal measured values within the whole lung of each case (grey line) indicate the smallest deviations at the left superior lung position (LS), slightly larger deviations at the left and right inferior sites (LI and RI) and at the right medial position (RM), and the largest deviations from minimal measured values at the right superior (RS) and left and right central lung positions (LC and RC). **(a)** data distribution, **(b)** deviation of the left superior position (LS) in comparison with min, **(c)** deviation of the left inferior position (LI) in comparison with min, **(d)** deviation of the left central position (LC) in comparison with min, **(e)** deviation of the right superior position (RS) in comparison with min, **(f)** deviation of the right medial position (RM) in comparison with min, **(g)** deviation of the right inferior position (RI) in comparison with min, **(h)** deviation of the right central position (RC) in comparison with min

The extents to which diatom concentrations of each lung position deviated from the lowest measured diatom concentration within each case are displayed in Fig. 4a. Since lung position LS did not have the lowest diatom concentration in every case, the case-specific differences to the respective lowest measured concentrations (grey line) were presented for each lung position individually (Fig. 4b-h).

Position LS showed the smallest deviation from minimal values, with the average variance being at only 0.31% (Fig. 4b). However, in 4 out of 25 cases – of which three were identified as extreme outliers – other lung positions exhibited lower diatom concentrations. The second lowest average deviation (3.2%) was found for LI, which included two cases presenting the overall lowest concentration at this position, but also one case with a very high deviation of 12.5% (Fig. 4c). In three cases, the lowest overall concentration was located at position RM and in one case at position RI (Fig. 4f, g). Respective average variances were 3.5% and 4.4% with both positions showing high deviations in five cases. Average deviation at position RS (6.5%) was only slightly higher than at position LC (6.3%), but by far the highest average deviation was displayed by position RC with 9.4% (Fig. 4d, e, h), with none of these positions reaching an overall minimal concentration in any case. Regarding the variability of concentration values, position RC showed unusual deviations in nine cases, whereas six and seven extreme deviations were observed at positions RS and LC.

### Effects on L/D-ratios

**Fig. 5:**
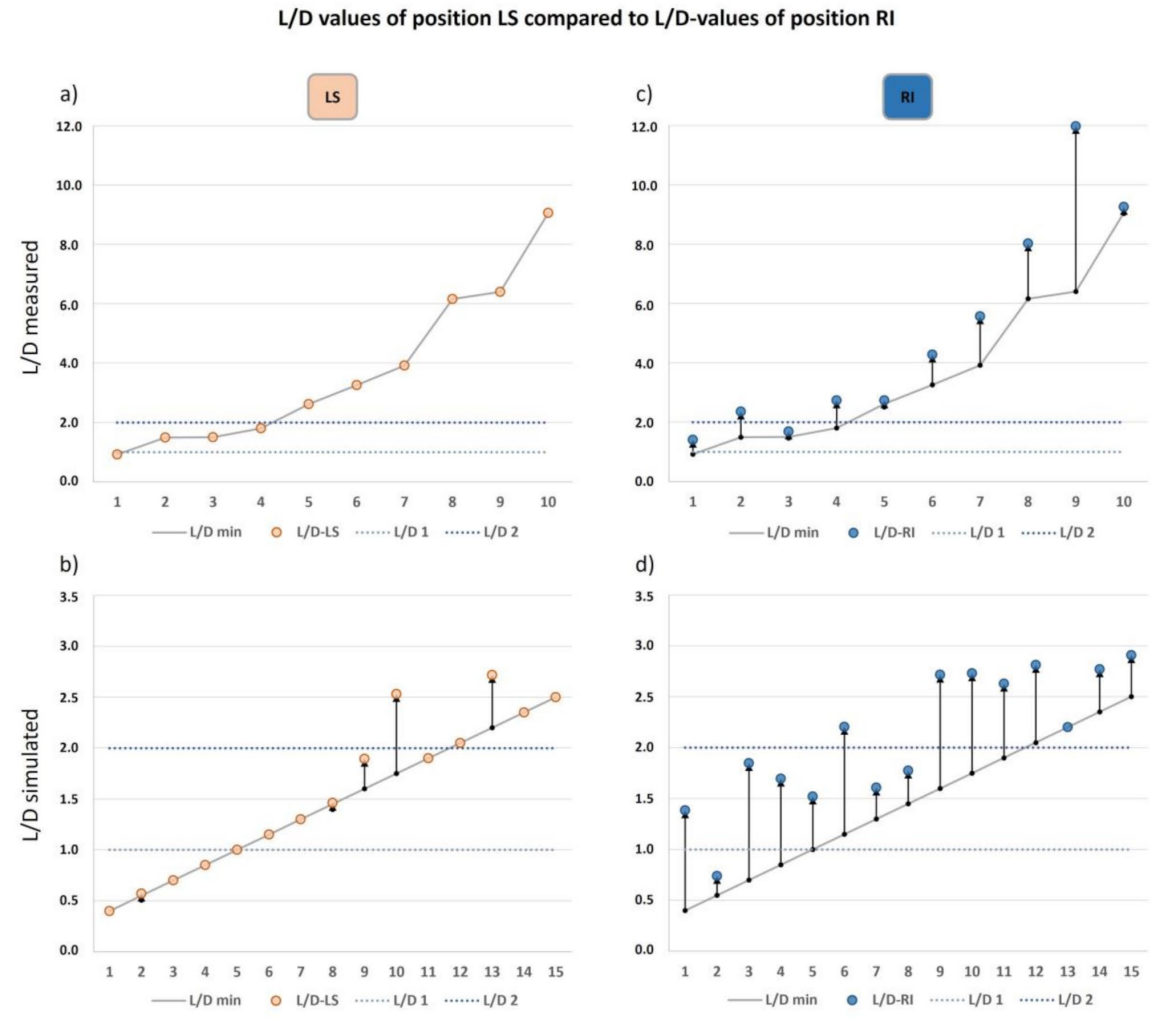
Comparison of L/D-values calculated with different lung positions in enumerating order. **a**) measured L/D-values with the left superior lung position LS, representing the minimal L/D-value line as all cases showed the least diatom concentration at this position, **b**) simulated L/D-values for the left superior lung position LS, representing the minimal L/D-value in 10 cases and an increase in 5 cases, **c**) measured L/D-values for the right inferior lung position RI, indicating an increase of the L/D-values in all cases, **d**) simulated L/D-values for the right inferior lung position RI, representing one minimal L/D-value and an increase in 14 cases

In all ten cases that allowed true L/D-ratio calculations as provided with water samples from the drowning site, position LS displayed the lowest concentrations and therefore represents the minimal L/D-ratio line (Fig. 5a). Six of these cases showed L/D-ratios ​​above 2 and were classified as drowning cases, whereas 3 cases could be diagnosed as probable drowning cases with ratios between 1 and 2. However, one case showed a ratio just below 1 and did not allow a reliable diagnosis by means of L/D-ratio determination. By contrast, calculating these L/D-ratios as an example with the diatom concentrations of position RI would result in higher ratios for all ten cases and distort the reliability of the diagnoses in three cases. Two additional cases would also reach ratios above 2 and be diagnosed as drowning cases, whereas the diagnosis of the uncertain case would be altered of being probably drowning (Fig. 5c). Similar to position RI, the same effects would apply to L/D-ratio calculations with diatom concentrations of other lung positions, as well as the L/D-ratio simulations performed for the 15 cases without drowning water samples, which led to the following results: With the diatom concentrations of position LS, again ten cases represented the minimal L/D-ratio line. However, 5 cases had their minimal diatom concentration at a different lung position and therefore displayed an increased ratio (Fig. 5b). On the other hand, simulations with position RI would have increased the ratios of nearly all these cases with probable falsification of the diagnosis in 14 out of 15 cases, except for the case with the least concentration at this position (Fig. 5d).

## Discussion

This study implicated significant differences in the accumulation of diatoms within the lungs of drowning victims. These results are addressed below, while also considering general organ structure and respiratory function.

### Implications of lung anatomy and flow dynamics on diatom distribution

While pulmonary anatomy, ventilation and perfusion of the lung have been well studied by various techniques, such as computer tomography and scintigraphy with radioactive substances [17, 18, 19, 20], it still remains unclear how aspired water behaves, disperses and affects air redistribution within the lung during a drowning incident.

The lung consists of five lobes, three on the right and two on the left side. Due to this anatomical characteristic, the left lung is smaller and lighter in weight than the right lung [21, 22]. However, the superior lobe of the left lung is the largest lobe and combines 5 of the 9 left bronchopulmonary segments, 3 upper lung segments (apical, posterior and anterior) and 2 segments of the lingula (lateral and medial). By contrast, the superior lobe of the right lung only combines 3 of the 10 right segments (apical, posterior and anterior), with the right medial lobe holding 2 segments (lateral and medial), whereas the left and right inferior lobes include the remaining basal segments, five on the right side and four on the left [23, 24].

During normal breathing, the superior regions are inflated first. However, due to their lower elasticity, the larger transpulmonary pressure at the apex and the effect of gravity, they are slightly less ventilated than inferior regions [17, 25], while perfusion is often reduced at the superior tips of the lung [26, 27]. Immersion of a body in water up to the neck is known to affect some of these factors, as compression by hydrostatic pressure causes a cranial shift of the diaphragm and enhances venous returns, which results in diminished pulmonary compliance, airway obstruction and a decrease of total lung capacity and expiratory residual volume [28, 29, 30]. It may therefore be assumed that these factors above also apply during breath holding under full submersion, until the continuous increase of CO_2_ partial pressure in the blood ultimately induces the inhalation of water [31, 32]. However, there is a lasting lack of experimental data concerning the effects and dispersion of inhaled water in this stage of drowning [33]. In this aspect, the present study may provide further information.

Assumed that the measured diatom concentrations in different lung areas of drowning victims roughly correlate with the volume of inhaled water and its dispersion within the lung, the results of the present work clearly indicate that water aspiration largely follows the pathways and dispersion patterns of regular lung ventilation during air breathing.

This is also in agreement with the anatomical structure of the bronchial tree in that diatoms concentrate in the lung’s central areas rather than in its peripheries, simply because the volume of passing water is highest there. Analyzed in more detail, this also explains and confirms the finding that diatom concentrations were found higher in the right central region than in the left, since the right main bronchus is more voluminous, has a steeper axis and divides proximally into lobal bronchi, which results in a higher absorbance of air, particles and/or water [34, 35]. Similar circumstance likely also accounts for the higher diatom concentrations in the right superior region. The bronchus of the right upper lobe already subdivides at a distance of 1–2.5 cm from the tracheal bifurcation [35], whereas the bronchial branches of the right medial and inferior lobe, as well as those of the left superior and inferior lobes all subdivide from their main bronchi at distances of about 5 cm from the tracheal bifurcation, accounting for a more delayed fill-up with water in the periphery as well as the left superior lobe, as aspired water might first reach segments of the lingula before reaching apical (superior) segments.

The side asymmetry of diatom deposition in drowning lungs is further underpinned by the findings on the deviations of minimal measured diatom concentrations, which appear to have smaller fluctuations within the left lung and tend to have higher extreme maxima on the right side. This asymmetry is also evident when looking at diatom amount fluctuations between cases at individual lung positions. By far the most and highest deviations were observed for central areas and the right superior lung position, consequently accounting for high average deviations at these positions. Although the right medial and inferior position demonstrated lower average deviations from minimal measured values, the average deviation of the left inferior position was substantially smaller than those of the right side. However, the left superior position (LS) exhibits the smallest deviations overall, even though not all cases hold the minimal concentration at this position. This could have several reasons. Although principally acting on all lung positions, environmental and physio-pathological factors such as water tonicity, temperature, redistribution of residual air, general fitness and medical conditions (especially of the lung) could influence the dispersion of aspirated water. Moreover, also the body position during a drowning incident must be considered to exert additional influence [31], as well as the fact that higher diatom concentrations in the water and larger diatom valves might obstruct smaller bronchioles, possibly leading to lower diatom concentrations in the periphery of the lung [36, 37].

All this does not diminish but rather supports the conclusion that the tip of the left lung (position LS) is the site of least diatom accumulation and the consistently smallest variations during a drowning incident, and even when case specific variations are taken into account, position LS can be clearly confirmed the most suitable sampling site for diatom-based forensic drowning diagnosis.

### Considerations on L/D-ratios

The L/D-ratio is certainly a valuable tool to provide supportive evidence in the confirmation of drowning events. Ratios above 2 can be held as proof of active water aspiration, making causes of death other than drowning unlikely. Nevertheless, limitations remain regarding the absolute reliability of L/D-ratios. Although passive infiltration of diatoms and other microorganisms into the lung is rather unlikely without active circulation [38, 39, 40, 41], recent studies have confirmed the possibility of diatom accumulation in tissues via the gastrointestinal tract [42]. Therefore, despite the limited uptake of large diatom valves [16, 43] and potential phagocytosis, L/D ratios close to or just above 1 must still be considered a grey area with less accuracy [13, 15]. Thus, it appears important to have an improved strategy to validate and expand L/D-ratio based diagnosis. The present study suggests that this task can be accomplished by measuring diatom abundances according to standardized criteria at lung positions with the least risk of overestimation. Results show that the superior tip of the left lung (position LS) is best suited in this regard, as all other tested lung positions yielded higher probabilities of overestimation, particularly those in central areas.

This appears well secured for all 10 cases with available reference samples of drowning water. L/D-ratio calculations with the diatom concentrations of position LS confirmed drowning as cause of death in six of these cases (L/D-ratio > 2) and supported the classification ‘probably drowning’ in three further cases with ratios between 1 and 2, whereas only one case, with a value slightly below 1, did not permit clear diagnosis. L/D ratios exemplarily calculated for position RI were in all cases higher than those for position LS and implied overestimations. Even if an increase is negligible for the cases with previous calculated L/D-ratios > 2 with position LS, since further increases of the ratio would not have altered the diagnostic outcome [40], they are particularly important at lower ratios. When L/D-ratios were calculated with position RI, two of the cases previously classified as ‘probably drowning’ would have reached ratios above 2 and the one uncertain case a value above 1, which would have led to different diagnoses. A similar picture was found for the cases without reference water samples. L/D-ratio simulations for position LS produced overestimations in five cases, although with potential relevance in only in three of these cases, since the other two cases just slightly deviate from the minimum. By contrast, L/D-ratio simulations for position RI would lead to misdiagnoses in 14 out of 15 cases.

Interestingly, support for further diagnostic ‘fine tuning’ could derive from the one case with a true drowning medium sample, which was classified as uncertain based on its L/D-ratio at position LS. This case was special because the expression of several drowning signs was clearly evident, which allowed no other conclusion than drowning. Thus, some factors must have hampered diatom uptake in the lung, entailing a falsely low L/D-ratio. As autopsy revealed pill residues in the stomach (no toxicological analysis performed), there could have been pharmacological influence on respiratory physiology and consequently the amount of inhaled water. Alternatively, despite two independently taken drowning water samples, mistakes could have also occurred during the extraction and handling of the reference water, with potentially great influence on the outcome of this type of analysis [12].

However, much more likely this might be a consequence of the rigorous attempt to minimize wrong positive results. In the pursuit to identify the lung region with the absolute minimum of diatoms, the chance for false negative results inevitably increases. Thus, for L/D-ratios close to 1, the diagnosis does not have to be concluded from this lung region alone and other lung regions (potentially with higher diatom concentrations) may be considered for additional investigation.

## Conclusion

The analysis of seven different lung positions in 25 drowning cases confirmed that the superior tip of the left lung (position LS) is the site with the lowest potential to accumulate diatoms during drowning, and also the site with the lowest average deviation from minimal diatom concentrations in the human lung after drowning. Consequently, position LS may therefore be regarded as best suited for tissue sampling to determine L/D-ratios for the diagnosis of drowning. However, relevant individual variability (as common in human studies) and the occurrence of outliers suggests that LD-ratio based diagnosis needs not be limited to position LS solely. Particularly in cases with L/D-ratios near the threshold value of 1, other lung regions with potentially higher diatom concentrations could also be considered.

A limitation of the present study is that it only included cases which have already been confirmed as drowning by autopsy. Consequently, the obtained results do not allow a reliable conclusion on diatom distribution in the lungs of non-drowning cases. However, the experimental applied procedure seems suitable for further studies to investigate the extent of passive diatom infiltration and accumulation in an animal model.

## Electronic supplementary material

Below is the link to the electronic supplementary material.

Supplementary Material 1

## Data Availability

All data generated or analyzed in course of this study are included in the article.
