# Supplementary material for: Quantitative investigation of diatom dispersion in lung tissue of confirmed drowning incidents
Source: Int J Legal Med. 2025 Feb 8;139(4):1597–607. doi: 10.1007/s00414-025-03441-1 (PMC12170756; doi:10.1007/s00414-025-03441-1)

#### Supplements

Table S1 Projected diatoms-per-gram-values and calculated percentage of the total of all positions in seven different lung positions of 25 drowning cases. LS left superior, LI left inferior, LC left central, RS right superior, RM right medial, RI right inferior, RC right central.

| **Case no.** | **Diatoms per gram** | | | | | | |  | **Diatom concentration in % calculated of the total of all positions** | | | | | | |
| --- | --- | --- | --- | --- | --- | --- | --- | --- | --- | --- | --- | --- | --- | --- | --- |
|  | **LS** | **LI** | **LC** | **RS** | **RM** | **RI** | **RC** |  | **LS** | **LI** | **LC** | **RS** | **RM** | **RI** | **RC** |
| 1 | 5642 | 8074 | 12040 | 10279 | 8407 | 8580 | 12525 |  | 8.6% | 12.3% | 18.4% | 15.7% | 12.8% | 13.1% | 19.1% |
| 2 | 2353 | 2879 | 11471 | 6228 | 3627 | 8141 | 20621 |  | 4.3% | 5.2% | 20.7% | 11.3% | 6.6% | 14.7% | 37.3% |
| 3 | 2705 | 4842 | 4358 | 5472 | 5699 | 3523 | 5604 |  | 8.4% | 15.0% | 13.5% | 17.0% | 17.7% | 10.9% | 17.4% |
| 4 | 13415 | 13078 | 18428 | 16064 | 12917 | 17330 | 19508 |  | 12.1% | 11.8% | 16.6% | 14.5% | 11.7% | 15.6% | 17.6% |
| 5 | 5480 | 6379 | 6934 | 6057 | 5935 | 6460 | 7919 |  | 12.1% | 14.1% | 15.4% | 13.4% | 13.1% | 14.3% | 17.5% |
| 6 | 6234 | 9512 | 9675 | 8677 | 6757 | 7251 | 11934 |  | 10.4% | 15.8% | 16.1% | 14.5% | 11.3% | 12.1% | 19.9% |
| 7 | 4166 | 5641 | 6812 | 12913 | 8698 | 6330 | 12981 |  | 7.2% | 9.8% | 11.8% | 22.4% | 15.1% | 11.0% | 22.6% |
| 8 | 1003 | 1329 | 3004 | 1556 | 1598 | 1923 | 2591 |  | 7.7% | 10.2% | 23.1% | 12.0% | 12.3% | 14.8% | 19.9% |
| 9 | 4704 | 6430 | 5288 | 6317 | 6112 | 5820 | 6235 |  | 11.5% | 15.7% | 12.9% | 15.4% | 14.9% | 14.2% | 15.2% |
| 10 | 10237 | 10159 | 13505 | 12005 | 13236 | 12434 | 13559 |  | 12.0% | 11.9% | 15.9% | 14.1% | 15.5% | 14.6% | 15.9% |
| 11 | 20508 | 28281 | 29608 | 67384 | 17323 | 29423 | 20460 |  | 9.6% | 13.3% | 13.9% | 31.6% | 8.1% | 13.8% | 9.6% |
| 12 | 4769 | 10507 | 15304 | 34514 | 14473 | 6770 | 57604 |  | 3.3% | 7.3% | 10.6% | 24.0% | 10.1% | 4.7% | 40.0% |
| 13 | 620 | 649 | 842 | 684 | 689 | 698 | 838 |  | 12.4% | 12.9% | 16.8% | 13.6% | 13.7% | 13.9% | 16.7% |
| 14 | 6410 | 8129 | 8206 | 6889 | 6568 | 6544 | 9896 |  | 12.2% | 15.4% | 15.6% | 13.1% | 12.5% | 12.4% | 18.8% |
| 15 | 8571 | 9704 | 11337 | 15282 | 5928 | 9246 | 24431 |  | 10.1% | 11.5% | 13.4% | 18.1% | 7.0% | 10.9% | 28.9% |
| 16 | 7553 | 8179 | 11098 | 7728 | 7891 | 7883 | 12400 |  | 12.0% | 13.0% | 17.7% | 12.3% | 12.6% | 12.6% | 19.8% |
| 17 | 2325 | 2690 | 4969 | 4816 | 2651 | 3525 | 5868 |  | 8.7% | 10.0% | 18.5% | 17.9% | 9.9% | 13.1% | 21.9% |
| 18 | 238 | 270 | 292 | 263 | 436 | 330 | 380 |  | 10.8% | 12.2% | 13.2% | 11.9% | 19.7% | 14.9% | 17.2% |
| 19 | 1798 | 2649 | 3303 | 2833 | 2350 | 2467 | 2761 |  | 9.9% | 14.6% | 18.2% | 15.6% | 12.9% | 13.6% | 15.2% |
| 20 | 4242 | 3782 | 5957 | 4815 | 4007 | 3437 | 3875 |  | 14.1% | 12.6% | 19.8% | 16.0% | 13.3% | 11.4% | 12.9% |
| 21 | 1509 | 3709 | 2335 | 2340 | 3802 | 1981 | 1860 |  | 8.6% | 21.2% | 13.3% | 13.3% | 21.7% | 11.3% | 10.6% |
| 22 | 696 | 919 | 1063 | 1360 | 1033 | 1302 | 1413 |  | 8.9% | 11.8% | 13.7% | 17.5% | 13.3% | 16.7% | 18.2% |
| 23 | 881 | 1099 | 2208 | 1982 | 1524 | 1393 | 1521 |  | 8.3% | 10.4% | 20.8% | 18.7% | 14.4% | 13.1% | 14.3% |
| 24 | 2325 | 3496 | 2728 | 2619 | 2756 | 6139 | 2341 |  | 10.4% | 15.6% | 12.2% | 11.7% | 12.3% | 27.4% | 10.5% |
| 25 | 6369 | 7783 | 7465 | 7334 | 7100 | 12695 | 8556 |  | 11.1% | 13.6% | 13.0% | 12.8% | 12.4% | 22.2% | 14.9% |

Fig. S1 Diatom dispersion within the lung of 25 drowning cases.


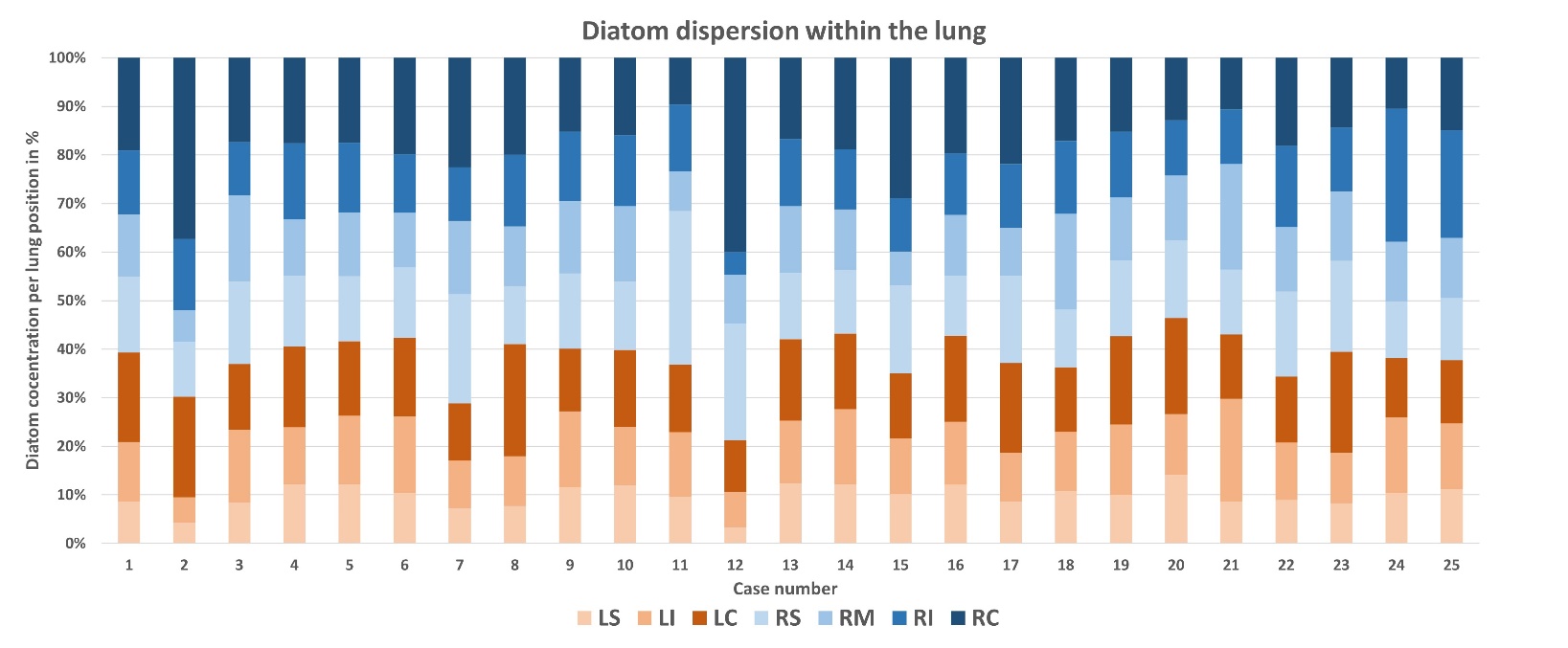

Supplement: Supplementary file 1 — Supplementary Material 1 [file 414_2025_3441_MOESM1_ESM.docx]
